# MIMIC-MJX: Neuromechanical Emulation of Animal Behavior

**Published:** 2025-12-02

**Authors:** Charles Y. Zhang, Yuanjia Yang, Aidan Sirbu, Elliott T.T. Abe, Emil Wärnberg, Eric J. Leonardis, Diego E. Aldarondo, Adam Lee, Aaditya Prasad, Jason Foat, Kaiwen Bian, Joshua Park, Rusham Bhatt, Hutton Saunders, Akira Nagamori, Ayesha R. Thanawalla, Kee Wui Huang, Fabian Plum, Hendrik K. Beck, Steven W. Flavell, David Labonte, Blake A. Richards, Bingni W. Brunton, Eiman Azim, Bence P. Ölveczky, Talmo D. Pereira

**Affiliations:** 1 Department of Organismic and Evolutionary Biology, Harvard University, Cambridge, MA, USA.; 2 Computational Neurobiology Laboratory, Salk Institute for Biological Studies, La Jolla, CA, USA.; 3 Neurosciences Graduate Program, University of California San Diego, La Jolla, CA, USA.; 4 Mila, Montréal, QC, Canada.; 5 School of Computer Science, McGill University, Montréal, QC, Canada.; 6 Biology Department, University of Washington, Seattle, WA, USA.; 7 eScience Institute, University of Washington, Seattle, WA, USA.; 8 Computational Neuroscience Center, University of Washington, Seattle, WA, USA.; 9 Department of Brain and Cognitive Sciences, Massachusetts Institute of Technology, Cambridge, MA, USA.; 10 Picower Institute for Learning and Memory, Massachusetts Institute of Technology, Cambridge, MA, USA.; 11 Molecular Neurobiology Laboratory, Salk Institute for Biological Studies, La Jolla, CA, USA.; 12 Department of Bioengineering, Imperial College London, London, United Kingdom.; 13 Howard Hughes Medical Institute, Cambridge, MA, USA.; 14 Department of Neurology and Neurosurgery, McGill University, Montréal, QC, Canada.; 15 Learning in Machines and Brains Program, Canadian Institute for Advanced Research, Toronto, ON, Canada.; 16 Montréal Neurological Institute, McGill University, Montréal, QC, Canada.; 17 Center for Brain Science, Harvard University, Cambridge, MA, USA.; 18 Kempner Institute, Harvard University, Cambridge, MA, USA.

## Abstract

The primary output of the nervous system is movement and behavior. While recent advances have democratized pose tracking during complex behavior, kinematic trajectories alone provide only indirect access to the underlying control processes. Here we present MIMIC-MJX, a framework for learning biologically-plausible neural control policies from kinematics. MIMIC-MJX models the generative process of motor control by training neural controllers that learn to actuate biomechanically-realistic body models in physics simulation to reproduce real kinematic trajectories. We demonstrate that our implementation is accurate, fast, data-efficient, and generalizable to diverse animal body models. Policies trained with MIMIC-MJX can be utilized to both analyze neural control strategies and simulate behavioral experiments, illustrating its potential as an integrative modeling framework for neuroscience.

## 1 Main

The nervous system evolved to control complex bodies in dynamic and uncertain environments. Studies on neural control of movement typically break the system down into manageable modules, whether functional and/or anatomic, and probe these in isolation [1–4]. While such reductionist approaches have enabled our current understanding of complex sensorimotor control, holistic alternatives that embrace the complex interplay between the brain, body, and environment [5–8] are ultimately required.

Successfully embracing this integrated view necessitates new tools for capturing, modeling, and simulating feedback interactions between neural and biomechanical systems in the context of natural behaviors. It is now possible to record the kinematics of natural behaviors in great detail using off-the-shelf cameras and easy-to-use software tools [9–15]. This progress enables routine measurements of detailed postural dynamics, but kinematic descriptions by themselves do not reflect the output of the brain’s control system. Instead, the nervous system operates on muscles which actuate a complex biomechanical body in rapid feedback with real-world physics.

Leveraging these advances to probe the neural control of movements and behavior will require incorporating the brain’s control of the biomechanical body. Artificial Neural Networks (ANNs) have been widely adopted as tractable and expressive substrates for modeling nervous systems *in silico*. Making practical use of ANNs as models of neural systems will require explicit multi-scale alignment to identifiable and measurable components. Work in this area is accelerating, with efforts directed at designing increasingly biologically realistic ANNs [16, 17]. For organisms with mapped connectomes, such as *C.elegans* [18] and *D.melanogaster* [19], using whole-brain wiring diagrams to constrain network architectures offers a path toward constructing mechanistic models whose connectivity is grounded in real circuit structure, enabling hypotheses that link neural circuits to embodied control in behaving animals.

Connecting such models of neural control to recorded kinematic trajectories will require modeling how the biomechanics of the body, together with the physics of the environment, generate forces that result in coordinated movement. Recent work has pioneered the use of physics simulation to model the biomechanics of complex behavior in diverse animals, including in rats [20, 21], flies [22–25], worms [26–30], and mice [14, 31, 32]. Unlike kinematics, however, motor commands and force production are not readily observable. Forces which actuate the body must be inferred since joint torques can not be directly measured in settings where animals may behave freely. This creates a significant challenge to align biomechanical simulation with behavioral data.

A separate line of work in computer graphics [33], robotics [34] and trajectory forecasting [35] devised approaches that use deep reinforcement learning (DRL) to train neural controllers capable of producing motor commands that mimic reference kinematic trajectories, a task commonly referred to as “motion imitation”. Building on this idea, a recent system called MIMIC [21] demonstrated that motion imitation can be successfully applied to a rat body model. However, because MIMIC was not well supported as open-source software and sufficient parallelization of its physics environments requires access to high-performance computing clusters with substantial CPU capacity, the system’s accessibility was substantially constrained. To address these deficits, we develop **MIMIC-MJX**, an open-source framework that builds on this motion imitation approach, aiming to democratize neuromechanical behavioral emulation from kinematics. MIMIC-MJX generalizes MIMIC’s approach to body models from different species (including muscle-based actuation) and greatly simplifies its deployment by leveraging MuJoCo XLA (MJX) [36] for massively parallel physics simulation on a single GPU, alongside ANN training.

MIMIC-MJX implements an end-to-end pipeline in JAX [37] that takes 3D pose tracking data as input, aligns it to biomechanically realistic body models, and trains ANNs to control these bodies within embodied physics simulation environments by learning to mimic the input kinematic trajectories. We show that MIMIC-MJX is accurate, fast, and efficient, making it accessible to academic labs without the computing resources available to industry researchers. We demonstrate its generalizability by applying it to a diverse set of scenarios: a wide range of species, under freely moving and restrained experimental settings, and using both previously published and novel biomechanical body models (a rat, fruit fly, mouse arm, stick insect, and worm). We validate and demonstrate the utility of our trained controllers by recapitulating results on gait kinematics and behavioral representation learning, and by leveraging their reuse as low-level controllers in a new task environment to enable simulated experimentation. Finally, to facilitate its adoption and further development, we have made our framework available as open-source software, along with all datasets, trained models, and documentation in the project page at: https://mimic-mjx.talmolab.org.

## 2 Results

### 2.1 MIMIC-MJX is a complete pipeline for training neuromechanical control models from motion data.

**Fig. 1:**
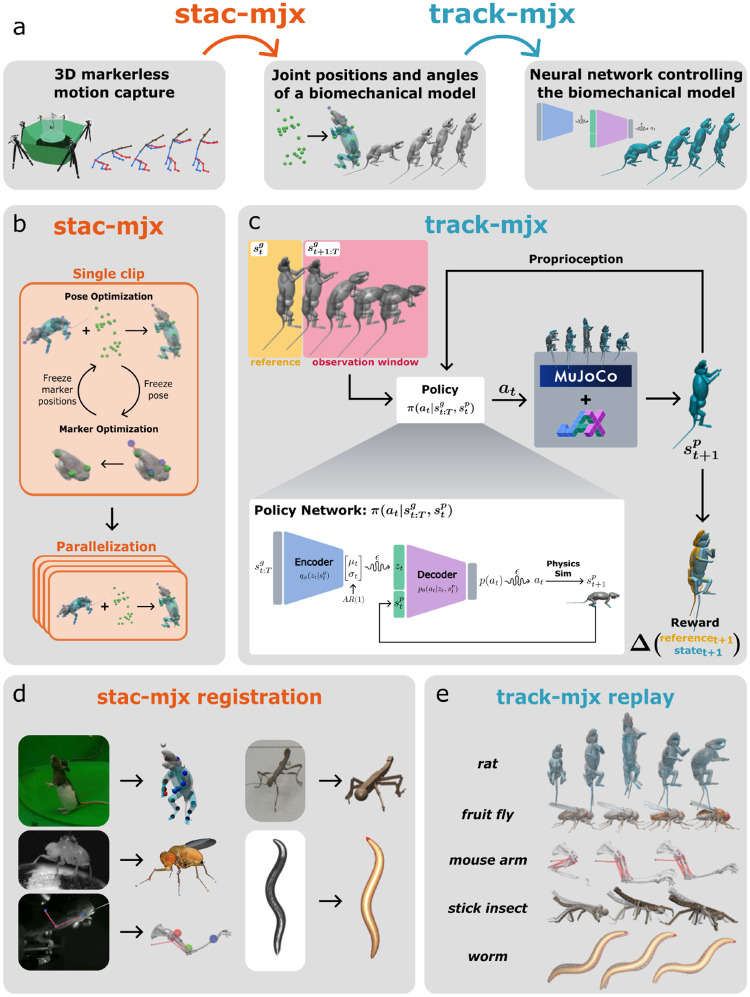
MIMIC-MJX is a complete pipeline for training neuromechanical control models from pose tracking data. **a**, Diagram of MIMIC-MJX at a high level **b**, Diagram of the **stac-mjx** module **c**, Diagram of the **track-mjx** training scheme **d**, **stac-mjx** registration is generalizable across arbitrary body models **e**, Rendered trajectories of **track-mjx** controlled agents (colored) and **stac-mjx** reference motion (gray)

The input to MIMIC-MJX is pose tracking data along with a MuJoCo-compatible biomechanical body model describing the animal’s kinematic chain, actuators that can receive control signals to elicit movement, and optional sensors (e.g., touch) for richer sensory feedback. Given these, **stac-mjx** is used to estimate calibrated joint-angle trajectories from the pose data via inverse kinematics. Then, **track-mjx** trains a neural controller that computes the motor control signals necessary to realize the tracked kinematic trajectories with the biomechanical body model (Fig. 1a).

In the first stage, **stac-mjx** implements a GPU-parallelized version of the Simultaneous Tracking and Calibration (STAC) algorithm for marker calibration and inverse kinematics [38]. It takes 3D pose tracking data as input and uses these to find the optimal configuration of joint angles of a given MuJoCo body model that is most consistent with the keypoint trajectories in the data (Fig. 1b). Because pose tracking data is often biased towards tracking surface landmarks rather than the internal skeletal structure, **stac-mjx** first performs marker registration to map keypoints to locations relative to a body part, grounding its position to the body model pose. Our approach is robust to noise in the tracked keypoints, and does not assume that every joint in the body model has a corresponding keypoint.

To facilitate the application of this system to new body models and tracking data, we developed a graphical user interface (GUI) that enables interactive selection of correspondences between keypoints and their most appropriate counterpart in the body model, while also solving for an optimal geometric transformation (scaling, rotation, translation) to account for coordinate system differences in the data relative to the model (Supplementary Fig. 1).

**stac-mjx** speeds up the inverse kinematics process by parallelizing over temporal segments of the input pose tracking data. It is capable of processing data at ~300 frames per second on an A100 GPU, enabling full inverse kinematics of an hour-long session of pose tracking in just 20 minutes (Supplementary Fig. 2). The output of **stac-mjx** is stored in a portable HDF5 file containing the solved body configuration for all frames and derived features, including Euclidean coordinates, joint angles, and velocities.

In the second stage, **track-mjx** receives the postural trajectories registered to the body model as input, and optimizes a neural controller to actuate the body model and “track” the movements of the input reference trajectories (Fig. 1c).

The neural controller is an ANN policy network trained via deep reinforcement learning (DRL) to maximize a composite reward function capturing the similarity between reference trajectories and reproduced trajectories, using both allocentric and egocentric representations of pose: global position, global quaternion, joint angles, and end effector positions (see Methods for details).

The ANN is structured as an encoder-decoder architecture to facilitate reuse in downstream tasks. The encoder receives a window of future timesteps of joint angles in the reference trajectory and compresses these into a Gaussian-regularized stochastic latent vector that represents the “motor intention” of the agent. The intention is then decoded by another ANN module, which combines this intention with sensory inputs from the current state of the environment to compute the most appropriate action for the next time step. The action output parametrizes a probability distribution over actuator control signals specific to the body model and can represent any number of degrees-of-freedom (DOFs) or actuator type present in the body model (e.g., direct torque-control, position-control, or muscles).

To train the ANN policy, **track-mjx** uses the Proximal Policy Optimization (PPO) learning algorithm due to its effectiveness in continuous control policy learning [39]. **track-mjx** optimizes the ANN via PPO in batches of “rollouts”—sequences of consecutive timesteps of the agent-environment interaction loop in which the policy attempts to track a given reference trajectory. These batches comprise parallel simulations whose experiences are gathered to compute synchronous gradient updates to the ANN weights.

**track-mjx** implements this approach to learning a neural controller from reference trajectories with abstractions to generalize to any body model (Fig. 1d). It uses a composable configuration system through Hydra [40] for experimentation and supports extensive logging of the optimization trajectory through Weights & Biases [41], enabling granular visibility into individual reward terms, system performance, rollout visualizations (Fig. 1e) on held-out data and other metrics that facilitate troubleshooting and afford crucial insights when applying this system to new data.

Together, this system comprises a full-featured toolkit for neuromechanical emulation that eases the technical barriers to implementing DRL and physics simulation with GPU acceleration.

### 2.2 MIMIC-MJX accurately registers and reproduces observed keypoint sequences from real data.

**Fig. 2:**
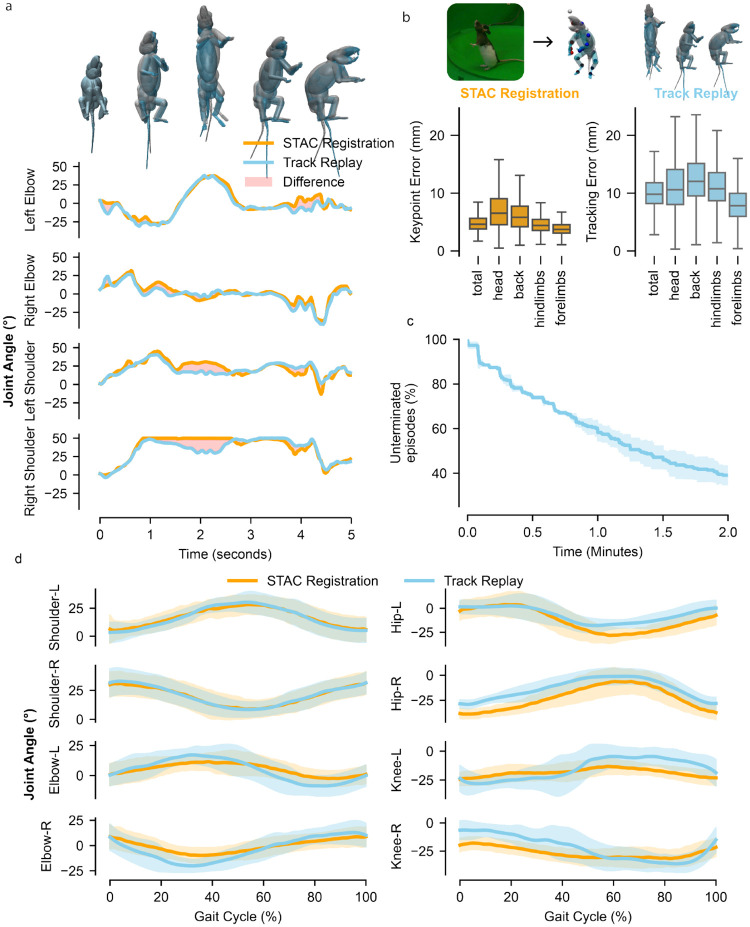
MIMIC-MJX accurately registers and reproduces observed keypoint sequences from real data. **a**, An example of a rollout of the rat tracking rearing behavior. Top: representative rendered frames of the biomechanical model during a roll-out showing the reference (gray) and reproduced (blue) trajectories. Bottom: joint-angle traces for the left and right shoulders and elbows over time; **stac-mjx**-registered reference (orange) and **track-mjx**-reproduced (blue) trajectories are overlaid, with their difference highlighted in the shaded region (pink). **b**, Keypoint error distributions between **stac-mjx**-registered reference and 3D pose tracking landmarks (orange) and between **track-mjx**-reproduced trajectories and **stac-mjx**-registered reference (blue). Errors are grouped by body region (forelimbs, hindlimbs, back, head) and total error. **c**, Evaluation of continuous tracking performance on a held-out dataset, measured as the percentage of episodes that remain unterminated as a function of elapsed time (minutes). The shaded area denotes the variance of the performance across 3 evaluations. **d**, Phase-normalized gait kinematics across multiple locomotion cycles. Joint-angle trajectories (degrees) for left/right shoulders and elbows (left panel) and hips and knees (right panel) over the gait cycle (0–100%); **stac-mjx**-registered (orange) and **track-mjx**-reproduced (blue). Shaded bands indicate variability across strides (n = 18 bouts).

We first applied MIMIC-MJX to train a neural controller for a virtual rat model, using a dataset of diverse freely-moving rat behavior as reference trajectories. Behaviors were classified as walking, rearing, and grooming, and segmented into 5-second clips [21]. ANNs trained with **track-mjx** reproduced trajectories accurately, closely following the observed reference sequences registered by **stac-mjx** as measured by the alignment of joint angles between reference and generated trajectories (Fig. 2a).

Summarized across the entire held-out dataset, we found that **stac-mjx** and **track-mjx** achieved low registration and tracking error, respectively. Performance was quantified as the absolute Euclidean distance between predicted and target keypoints. **stack-mjx** showed low median registration error (~5 mm) relative to the pose tracking keypoint trajectories, while **track-mjx** likewise demonstrated low median tracking error (~10 mm) when evaluated against the **stac-mjx**-registered keypoints. This corresponds to less than ~5% of the adult rat body length, consistent with previous work [21], and reflects markerless keypoint localization error baselines [11, 42].

We next evaluated **track-mjx** trained controllers on their ability to track motion sequences at longer timescales than they were trained on. **track-mjx** tracking errors tended to slowly accumulate when the controller failed to completely recover from mistakes in the control sequence; at a threshold deviation distance from the reference pose, we terminated the episode and reset the environment by realigning the pose to the reference at that timestep. We tested our rat controller on an unseen session of kinematic data and measured the “unterminated episode percentage”—the fraction of episodes which were not yet terminated—over time, yielding the survival curve. The survival curve illustrated robust long-duration tracking: ~75% of episodes persisted at 0.5 minutes, and ~40% at 2 minutes, far beyond the training clip length of 5 seconds (Fig. 2c). This reflects our system’s ability to generalize to longer timescales and to faithfully simulate continuous control at the timescale of complex natural behavior sequences.

Finally, we assessed whether these error rates have an impact on downstream kinematic features used to characterize motor behaviors, such as locomotion. To do this, we segmented gait bouts of clear locomotion and phase-aligned the registered and generated movement sequences to a standard gait cycle. We found that MIMIC-MJX replicates expected patterns of locomotor coordination at each stage (Fig. 2d).

Together, these results reflect MIMIC-MJX’s ability to accurately reconstruct biomechanically plausible poses through motor actuation while maintaining the necessary temporal structure and joint-level dynamics for precise kinematic reproduction.

### 2.3 MIMIC-MJX generalizes to diverse animal models and experimental settings

**Fig. 3:**
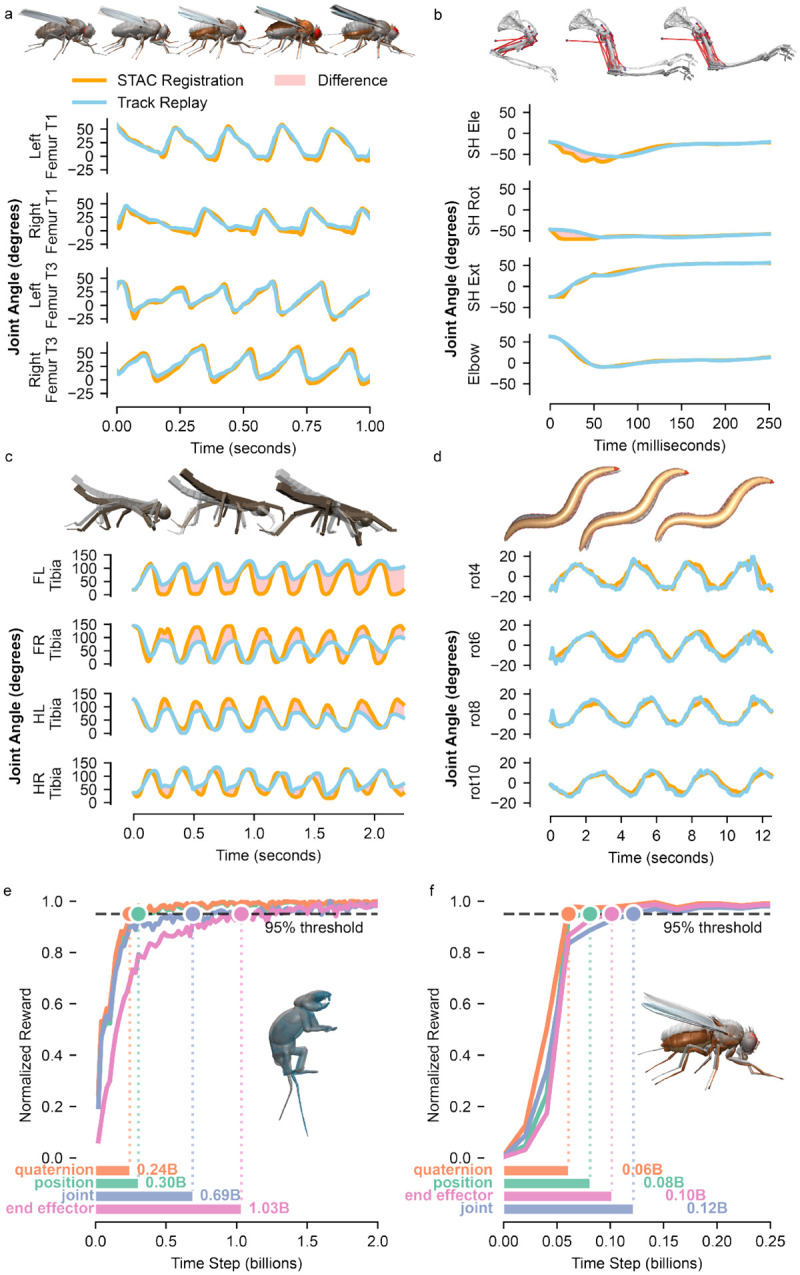
MIMIC-MJX generalizes to diverse animal models and experimental settings. **a-d** STAC registration and Track replay with pointwise differences highlighted from rollouts of a fly (**a**), mouse arm (**b**), stick insect (**c**), and worm (**d**). **e, f** Individual reward components of the rat (**e**) and fly model (**f**). The dashed line marks a 95% threshold of each term’s maximum observed value. The number of time steps required to reach the 95% threshold is shown.

We designed MIMIC-MJX to be flexible for diverse animal body models. To demonstrate this, in addition to our primary focus on the rat model, we successfully applied MIMIC-MJX to biomechanical models and pose tracking data from a fruit fly, a mouse arm, a stick insect, and a worm (Fig. 3a–d). Fruit fly, stick insect, and worm controllers were trained to track locomotor behavior, while the mouse arm controller tracked reaching trajectories. We demonstrate performance through the alignment of **track-mjx**-controlled joint sequences with **stac-mjx**-produced reference sequences. Configuration details for each implemented model are provided in the supplement (Supplementary Fig. 3).

Because our reinforcement learning reward is made up of multiple imitation accuracy reward components (i.e., alignment of root position, root quaternions, joint angles, and end-effector positions to the reference values), we studied each component’s learning trajectory during training for multiple body models. Root position and quaternion rewards can be thought of as “coarse” terms (global root alignment), and joint and end-effector rewards as “fine” terms (pose alignment). Composing these dense reward signals produces a consistent coarse-to-fine curriculum that is reflected in the learning dynamics of both rat and fruit fly (Fig. 3e,f). Early coarse alignment is achieved when the ANN first stabilizes global position and orientation. Fine kinematic refinement follows coarse alignment as the ANN learns to coordinate the many DoFs to match joint angle and end effector positions—a more constrained, higher-dimensional objective that requires additional experience. We observed degraded overall tracking performance when ablating the different tracking reward components (Supplementary Fig. 4).

### 2.4 MIMIC-MJX is fast and efficient

Making MIMIC-MJX an accessible framework for modeling the neuromechanical basis of behavior requires that training times and data requirements be compatible with iterative experimentation. For example, adding more diverse behavioral data, varying biomechanical or neural controller configurations, or extending it to a new species all require training the system from scratch. In previous work [21], this form of experimentation was limited by the speed of the physics simulation, a CPU-bound component of the pipeline that did not scale well with the computational resources available to academic researchers. Using JAX-based deep reinforcement learning and physics simulation, we bypassed this limitation, as demonstrated by its performance profile across different animal models and datasets.

We trained neural controllers for the same five biomechanical models (mouse forelimb, fruit fly, stick insect, rat, and worm) using their corresponding pose tracking datasets. To evaluate training efficiency, we quantified the number of data samples (in terms of environment timesteps—one agent-environment interaction loop) needed for a policy to attain near–asymptotic performance.

The DRL training runtime is primarily composed of two components: experience generation through the agent-environment loop and the actor/critic network gradient updates. For our physics-based environments, the dominant component by far is the experience generation. Therefore, we quantified training speed in terms of environment step throughput, measuring the number of steps required to reach 95% of the normalized test–set reward.

**Fig. 4:**
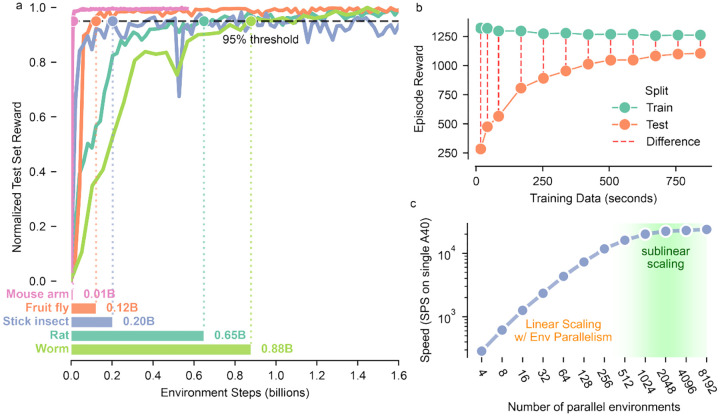
MIMIC-MJX is fast and efficient. **a**, Normalized test set reward versus environment steps for five biomechanical models (mouse arm, fly, stick insect, rat, and worm). Rewards are normalized to each model’s empirical maximum (unit peak = 1). The horizontal dashed line marks the 95% threshold; colored markers and matching vertical dotted lines indicate the first crossing for each model. The bars below report the number of environment steps (in billions) required to reach the 95% threshold. **b**, Mean episode reward as a function of training data size. Green: training split. Orange: held-out test split of 673 five-second clips. At each x (number of 5 s clips), the paired markers come from a single training run; red dashed connectors visualize the train–test gap. The y-axis reports the average reward over evaluation rollouts (the state trajectory produced during the environment-agent loop). **c**, Environment throughput on a single NVIDIA A40 GPU: steps per second (SPS) versus the number of parallel environments (log scale). Background shading denotes parallelism-bounded, balanced, and GPU-compute-bounded (not memory-bounded) regimes.

All agents reached the threshold in fewer than one billion steps: mouse arm ~0.01B, fruit fly ~0.12B, stick insect ~0.20B, rat ~0.65B, and worm ~0.70B (Fig. 4a). These results show that convergence time scales with biomechanical and behavioral complexity. The mouse arm, performing simple reaching trajectories with only four joints and nine muscle actuators, trained very quickly—converging in tens of millions of steps. The rat was trained on a richer behavioral repertoire compared to the more constrained reference behavior for the other animals, thus taking a longer time for training to converge. However, despite its reference data consisting of only undulatory locomotion, the worm was the slowest to train among the body models. This could be due to its large number of muscle-driven actuators (95), making the sensorimotor controller harder to learn [43].

To investigate generalization performance with respect to dataset size, we trained multiple controllers with varying amounts of training data. Beginning with 15 seconds of data, we trained on increasing durations of natural behavior until the training dataset totaled 845 seconds, or about 14 minutes. We report mean episode reward on both the training and test splits for each of these runs (Fig. 4b). Test rewards increased monotonically with data, and the train-test gap decreased steadily (red segments), indicating task generalization rather than memorization. Notably, most of the generalization gains were achieved within ~50 clips, and by 100 clips the test curve approached the training curve. This shows that the ANN can generalize to new trajectories with a high-quality training dataset on the order of only *minutes*. This underscores the data efficiency of the learned controller and shows its ability to reproduce new trajectories without retraining.

We benchmarked environment throughput on a single A40 GPU as a function of the number of parallel environments (Fig. 4c). Steps per second (SPS, the number of total environment steps completed per second), scaled linearly until GPU compute utilization became saturated. We noticed that for our environments, GPU memory (which constrains the total possible number of environments) is not a bottleneck for maximizing throughput on most GPUs.

We evaluated end-to-end throughput across commercial and consumer GPUs with various sizes of policy networks. We found that training converges within 6 to 20 hours on datacenter and consumer-grade GPUs, respectively, while datacenter-level CPU-only training takes over 30 hours (Supplementary Fig. 5). We also show predictable scaling when modulating the policy network size. The combination of high-throughput, single-device scaling through parallelism, and portability across widely available hardware lowers the barrier to entry.

### 2.5 MIMIC-MJX enables experiment simulation and neuromechanical behavioral analysis

A trained motion imitation policy yields useful modules that can be transferred to learn naturalistic solutions to downstream tasks. To transfer the pretrained policy, we repurposed the encoder-decoder architecture by replacing the encoder with a task-specific policy network. This new policy generated latent representations that served as inputs to the pretrained decoder, effectively grounding task behavior in the same latent motion space used during imitation training. Since the original encoder-decoder was trained with stochastic regularization, the resulting “motion intention” latent space was easy for the task-specific policy to explore, allowing effective control in novel environments. This produced new policies that were more closely aligned with naturalistic motion when solving the new task.

**Fig. 5:**
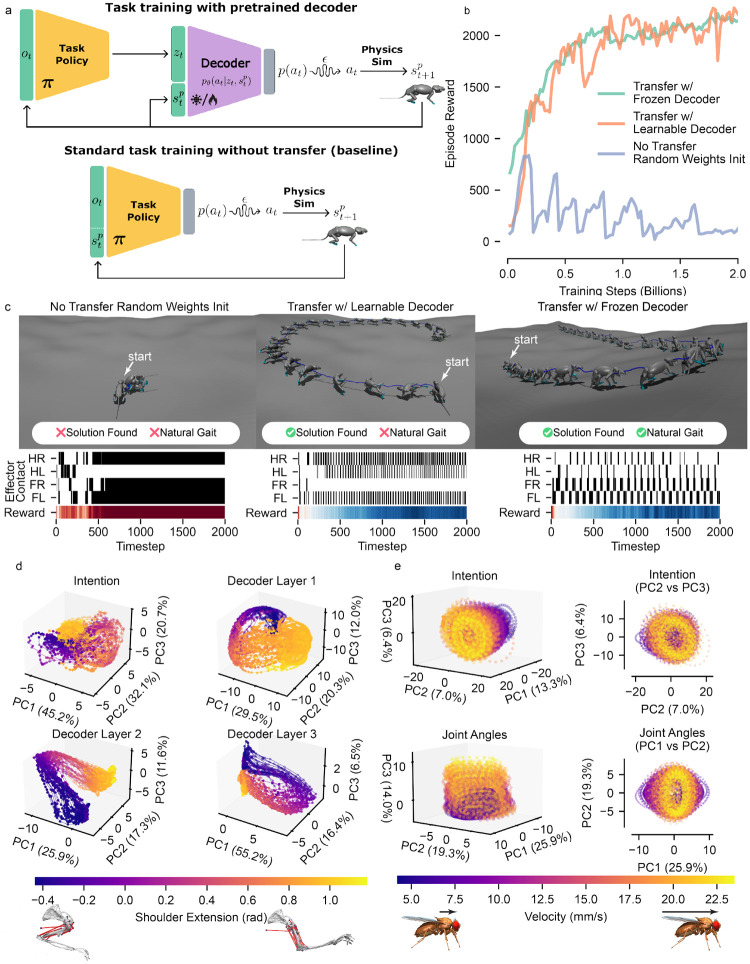
MIMIC-MJX enables experiment simulation and neuromechanical behavioral analysis. **a**, Diagram of MIMIC-MJX-trained decoder transfer to a downstream task **b**, Task reward curves for training by transferring a frozen decoder, transferring a learnable decoder, and without transfer (randomly initialized weights). **c**, Rendered rollout trajectories on the bumpy bowl escape task for policies trained with the three different configurations. Footfall raster plots and reward progression (below) reflect the differences in task performance of the three conditions. HR: Hind-right, HL: hind-left, FR: fore-right, FL: fore-left paws. A black line indicates a footfall for the given limb. Reward spectrum ranges from red (low reward) to blue (high reward). **d**, 3D PCA embeddings of mouse arm reaching trajectories, using representations from the intention space (top-left) and sequential decoder layers (top-right, bottom-left, and bottom-right). The axes show % variance explained for each PC. The colormap visualizes the extent of the shoulder extension. **e**, PCA of the fly during locomotion. 3D (top-left) and 2D (top-right) PCs of the neural controller’s intention space. 3D (bottom-left) and 2D (bottom-right) PCs of the stac-mjx-defined joint angles. The colormap visualizes the velocity of the locomotion.

We present the utility of motion imitation pretraining by transferring our rat policy network to solving a “bowl-escape” task [34]. Here, the agent had to navigate an environment of uneven terrain in the shape of a bowl, where the objective was to move away from the center of the bowl as fast as possible. We explored performing the transfer with (1) a frozen pretrained decoder, (2) a pretrained decoder with learnable weights, and (3) training the entire network on the bowl-escape task without imitation pretraining as a baseline (Fig. 5a).

We found that a randomly initialized MLP policy is unable to learn the task, while both policies leveraging the pretrained decoder were able to solve the task and reach the same reward (Fig. 5b). As shown by the footfall raster plots, the frozen transferred decoder qualitatively learned a more naturalistic locomotion strategy compared to the learnable decoder and the randomly initialized baseline (Fig. 5c). While the learnable decoder successfully solved the task, it did so through unnatural, “jittery” movement patterns, likely due to “catastrophic forgetting”—the phenomenon where learning a new task quickly results in the overwriting of previously learned solutions [44]. The randomly initialized baseline failed to solve the task entirely, producing neither natural behavior nor goal achievement. Notably, when we simplified the control problem by switching from torque to position actuators (which are generally easier to optimize), the randomly initialized policy solved the task, yet still exhibited unnatural gait patterns (Supplementary Fig. 6). This demonstrates that pretraining on the imitation objective using **track-mjx** enables the encoding of important priors for naturalistic behavior, which can subsequently be leveraged by higher-level controllers. Critically, freezing the decoder preserved these naturalistic priors while still enabling task learning, whereas allowing the decoder to adapt led to task success at the cost of behavioral realism.

In order to demonstrate how the geometry and dimensionality of the intention representation evolve as it is propagated through the decoder, we visualized the first 3 principal components (PCs) of the intention and latent activations of each hidden layer for all reaching tasks of the mouse arm (Fig. 5d). The latent intention encoded a compressed representation with 54.2% variance along its first principal component (98% across the first three PCs), indicating a strongly structured low-dimensional space. In the decoder, it was relatively higher dimensional in the early layers (54.8% explained variance in the first 3 PCs); however, by the final layer, it became lower-dimensional (78.1% variance explained in the first 3 PCs), consistent with the idea that motor activity resides in a low-dimensional subspace, potentially reflecting “motor synergies” [45]. We saw that a topological structure emerged in the later hidden layers of the network, where a toroidal structure with a noticeable topological invariant (or hole) represents the reaching cycle.

The learned latent space also encodes kinematically interpretable information. We visualized 3- and 2-dimensional PCA embeddings of the latent vectors during fly walking behavior, showing the low dimensional, cyclic manifold of the gait cycle (Fig. 5e top). Similar to previous work[46], each point represents the PCA embedding of a 30-timestep window of latent vectors, representing 0.06 seconds. In addition to the salient cycles along PCs 2 and 3, this manifold reflects a linear representation of gait velocity along the orthogonal PC1 axis, as shown by the color gradient denoting the average velocity of each clip smoothly changing along this axis. We performed the same analysis on the kinematic features (joint angles) acquired through **stac-mjx**, and found that it also results in the same structured embedding space (Fig. 5e bottom).

While these analyses serve as illustrative examples, they confirm that the latent space captures interpretable structure, highlighting the potential for applying more directed neural representational analysis techniques to the learned computations in the control policies [47, 48].

## 3 Discussion

Here, we present MIMIC-MJX, a fast and generalizable framework for modeling the neural control of movement during complex behaviors across species. Through its computationally efficient implementation and ease-of-use, MIMIC-MJX makes neuromechanical modeling accessible to a broader set of researchers.

However, the MJX physics simulator we rely on has some limitations, with future improvements currently in development by both the developers and open-source contributors. While we model the mechanosensory and proprioceptive sensory systems, other sensory modalities are not yet readily accessible. Vision, notably, is not fully supported, though we expect this to be addressed in the coming updates to the simulation framework. Auditory and olfactory modeling remain challenging due to their diffusive dynamics that are best captured through expensive fluid dynamics simulation [49], which are prohibitively expensive. Surrogate physical models and more efficient implementations are promising directions for overcoming these technical limitations [50]. MJX also does not scale well with large numbers of contacts with the environment. We circumvent this in MIMIC-MJX by only allowing end effectors (e.g., feet) to be in contact with the ground plane during simulation. Fortunately, the developers’ ongoing integration of Warp [51], another framework for high-performance simulation, into MJX will soon resolve this issue.

There are also several promising avenues of future development for MIMIC-MJX:

First, our training framework is limited to individual agents, making it unsuitable for exploring social behaviors and interaction dynamics. While the neural controllers produced by MIMIC-MJX can be used in multi-agent simulations, aligning them to social behavioral data requires additional work—particularly in developing reinforcement learning algorithms that integrate cues from other agents with low-level motor control [52].

Second, while we use DRL for optimization, we do not attempt to model realistic learning trajectories. Further work is necessary to simulate the biological and computational mechanisms of motor learning, though we note that our task transfer experiments demonstrate the capability of reusing the neural controller trained by MIMIC-MJX to achieve task-driven learning objectives with more naturalistic behavior (Fig. 5a–c).

Third, while we note that MIMIC-MJX is agnostic to specific ANN topologies and computational operators, we did not conduct extensive experiments with implementing ANN architectures intended to directly map to known biological neuroanatomy. It should be noted that this has been demonstrated in recent studies leveraging connectomic and functional data to constrain ANN-based models of the nervous system [53–57], albeit without biomechanical constraints.

Finally, a major difficulty in implementing this framework is the requirement of hand-crafted biomechanical body models with realistic actuators compatible with physics simulation. While tooling is being developed to facilitate the generation of body models [25, 58–60], this is a critical area for enhancing the utility of neuromechanical modeling, not just to broaden the species representation, but also to better capture the diversity of body morphologies across populations and developmental stages. The framework presented here demonstrates the urgent need for, and provides the computational scaffold to integrate, new high-throughput anatomical pipelines. The full power of MIMIC-MJX will be realized in future models incorporating more detailed anatomical and biomechanical models, moving the field beyond hand-crafted models toward comprehensive morphological datasets.

Future work will address these technical limitations and incorporate new features, but more broadly, this work is a critical step toward a new paradigm in which *in silico* simulation becomes an indispensable counterpart to *in vivo* experimentation. The full realization of this “Virtual Neuroscience Lab” extends far beyond a simple hypothesis-generation loop.

By creating fully configurable, embodied “digital twins,” we open the possibility of creating simulated test beds for both basic mechanistic discovery as well as simulation of disease states and putative therapeutics. For instance, we can move from virtual lesions to modeling the progressive degeneration of both neural and musculoskeletal systems, capturing the complex interdependencies of brain and body pathology in neurodegenerative diseases. This holds particular value for advancing foundational research into movement disorders, stroke and rehabilitation [61–64].

Furthermore, these models can serve as foundational tools for robotics and embodied AI. By uncovering the control architectures and learning algorithms that animals actually use, we can build more agile, efficient, and fault-tolerant robots. Ultimately, because our own intelligence co-opted the very circuits that evolved for sensorimotor control, these models of “embodied intelligence” may be a critical step toward artificial agents capable of more general, human-like functions.

## 4 Methods

### 4.1 Body Models

We used biomechanically validated models of the organisms we implemented into MIMIC-MJX:

- Rat (*Rattus norvegicus*): We use a biomechanical rat model developed in previous work [34], matching the structure of Long Evans rats. The model has 74 degrees of freedom (DoF), of which the Cartesian position of the model is represented by 3 degrees and the Cartesian quaternion is represented by 4 degrees. The remaining 67 DoF represent the joint angles relative to the parent’s body frame in the kinematic tree. The model has 38 controllable torque actuators, and we use a subset of the included sensors, which comprises (1) a velocimeter, (2) an accelerometer, (3) a gyroscope, and (4) force, torque, and touch sensors on its end effectors. The end effectors are chosen to be the four paws and the skull.
- Fruit fly (*Drosophila melanogaster*): We use a fruit fly model developed in previous work [25]. The model consists of 67 rigid body segments linked at 66 joints, yielding 102 degrees of freedom, and was reconstructed from high-resolution confocal microscopy of a female fly. Following their design, we remove the actuators for the wings, probiscis, and antennae when training for the walking imitation task, resulting in 61 torque actuators to drive the joints. The end effectors are chosen to be the pretarsal claws.
- Mouse arm (*Mus musculus*): A skeletal model of the mouse forelimb and estimations of muscle attachment points were provided based on light sheet microscopy data [60]. The model has 4 DoF in total: 3 DoF in the shoulder: elevation, rotation, and extension, and 1 DoF in the elbow. Our simplified model has 9 controllable parameters, which are the following Hill-type muscle actuators: triceps (long), triceps (lateral), biceps (long), brachialis, pectoralis (clavicular), latissimus, posterior deltoid, anterior deltoid, and medial deltoid. Muscle attachment points were refined, and muscle parameters were found to produce forces within the range of real mouse forelimb muscles 0.2–1.2N [32].
- Worm (*Caenorhabditis elegans*): We use a body model developed in previous work [26]. The model consists of 25 body segments linked by 24 joints. Each joint is actuated by 4 body wall muscles, dorsal left/right and ventral left/right – except for the tail, which lacks its ventral right muscle – making 95 body wall muscles. The model is constrained to 2D motion, i.e., only displacement in the XY plane and rotation along the Z axis. We also add anisotropic friction contact between the body model and the floor, which has been shown to be used by the worm to propel itself in agar [65].
- Stick insect (*Sungaya aeta*): We used a *Sungaya aeta* model consisting of 43 rigid body segments linked at 42 joints, resulting in 42 degrees of freedom. The model was constructed from a 3D scan obtained with the open photogrammetry platform *scAnt* [66]. The 3D mesh was preprocessed in Blender (Blender Foundation, Amsterdam, Netherlands; version 3.0), and then converted into an URDF model using *Phobos* [67]. The URDF model was converted into XML format and extended to an MJCF file using custom scripts.

For each of these models, we limit contacts between the model and the environment (a single ground plane) to only the end effectors to dramatically improve the throughput of MJX, which scales poorly with the number of contacts in the simulation. Because our imitation tasks only involve contact with the end effectors and implicitly constrain behavior to avoid self-collisions, these changes do not negatively impact the training results and are merely an optimization measure.

### 4.2 Kinematic Data

Markerless pose estimation was performed on multicamera video data to provide the necessary pose tracking data for the subsequent fit of inverse kinematics. The data are publicly available from their respective sources:

- Rat: We use a dataset collected in previous work [21] comprised of 842 5-second clips of freely behaving kinematic data, represented by 23 keypoints captured at 50 Hz. It was collected using DANNCE v1.3 [11] from multicamera video. It represents a wide range of behaviors, including various kinds of walking, rearing, and grooming.
- Fruit fly: We use a dataset collected in previous work [68], which uses a spherical treadmill to enable 3D kinematic tracking of the fruit fly at different locomotor speeds. This data was sampled at 300 Hz. The pose estimation was done with DeepLabCut [10] and Anipose [12]. To align the keypoint data (which uses arbitrary coordinate axes) to the body model using Cartesian coordinates, we first performed a Procrustes transformation to scale and rotate the average position of the keypoints in a single clip to the rest position of the fly body model. We then applied the transformation to all frames in the clip. Due to the data being collected on a spherical treadmill, the data was not exactly aligned to the ground plane. To address this, we then performed a second transform that aligned the tarsal-claw keypoints to the ground plane. Finally, we used the rotational velocity of the ball as a proxy for the fly’s linear and angular velocity in the xy-plane. The data was then run through stac-mjx. Then, the result was linearly interpolated to be sampled at 500 Hz so that there are 10 physics steps per control step.
- Mouse arm: A three-camera dataset was labeled using the CVAT annotation tool to generate ~10,000 labeled frames per camera. This network was then run on a video consisting of 46 successful water reaching trials [69]. Predictions of the pose of the right shoulder, elbow, and wrist were generated with a single animal pose estimation network using the U-Net architecture in SLEAP [13]. A calibration video was recorded by placing a ChArUco board in the view of all cameras. Calibration was performed using the SLEAP-Anipose package, which is a wrapper around Anipose to allow for easy integration of 2D pose data from SLEAP. Calibration was performed with the SLEAP-Anipose library, which uses OpenCV to perform iterative sparse-bundle adjustment. It locates the 2D points on the ChArUco board and then triangulates them into 3D. Then the reprojection error is calculated by projecting back into 2D to evaluate the 3D estimation. This reprojection error is then minimized iteratively using L-BFGS to improve the 3D estimates and provide reliable triangulation.
- Nematode worm: We use a dataset collected and analyzed in previous work [70, 71] containing five 6-hour videos of freely moving *C. elegans*. The videos were collected using a custom LabView program from a tracking microscope captured at 20 Hz. We downsampled the 500 centerline points to 25 points via linear interpolation. The dataset represents a repertoire of forward/backward locomotion, omega turns, and headswings from worms spontaneously behaving on a food plate. However, we limit training to clips of forward locomotion.
- Stick insect: We used a dataset collected on a flat and smooth treadmill that perpetually kept *S. aeta* inside the imaging volume of a five camera array (ORX-10GS-51S5C-C, Teledyne, USA; Lens: 2/3” 8mm f/2.8 16 Megapixel Lens, Computar, Japan); active 2D motion compensation enabled the recording of untethered 3D kinematics at 75 Hz. Marker-less pose estimation of 50 keypoints was achieved across all camera views using a single U-net architecture in SLEAP [13]. The network was pre-trained using synthetic data [72], and iteratively refined using 800 hand-annotated frames, selected from all five views. A calibration video was recorded by placing a checkerboard in view of all cameras. Camera calibration and triangulation were performed using Anipose [12].
  The extracted 3D coordinates were translated and rotated to align the XY plane of a body-fixed coordinate system with the treadmill plane. The 3D kinematic data was offset to account for the movement of the treadmill, estimated by tracking ArUco markers printed onto the belt surface using OpenCV, to obtain the absolute walking speed [73, 74].

### 4.3 stac-mjx Joint Marker Registration and Inverse Kinematics

stac-mjx implements a modified version of the Simultaneous Tracking and Calibration (STAC) algorithm [38], originally developed for system identification in robotics. The algorithm takes an iterative approach to solving inverse kinematics (“tracking”) and marker positions (“calibration”) simultaneously, formulated as the maximum likelihood problem $\sum_{m}{\text{min}}_{\theta_{m}}\left(\sum_{t=1}^{T}{\text{min}}_{q_{t}}\;\text{log}\;p\left(v_{t}^{m}|q_{t};\theta^{m}\right)\right)$, where $q_{t}$ represents joint angles, $\theta_{m}$ denotes marker site offsets, and $v_{t}^{m}$ are marker site positions. stac-mjx is an implementation of this algorithm designed for use with animal pose tracking, leveraging JAX and MJX for fast parallelization on GPU.

Our implementation is based on a previous implementation [21], which incorporated a warm start to inverse kinematics by beginning with optimizing the root body position only before proceeding to full body inverse kinematics. Then, individual parts of the model’s kinematic tree are optimized independently for greater accuracy. We incorporate these additions to our GPU-accelerated implementation. We use Projected Gradient Descent (PGD) to optimize the pose given the individual frame’s keypoints, using box constraints to limit joint angle ranges. For optimizing the marker positions, we use Stochastic Gradient Descent (SGD) given every frame’s keypoints and corresponding body model pose in joint angles. Algorithm 1 describes the process in detail. We include standard configuration parameters for **stac**-**mjx** in the code documentation.


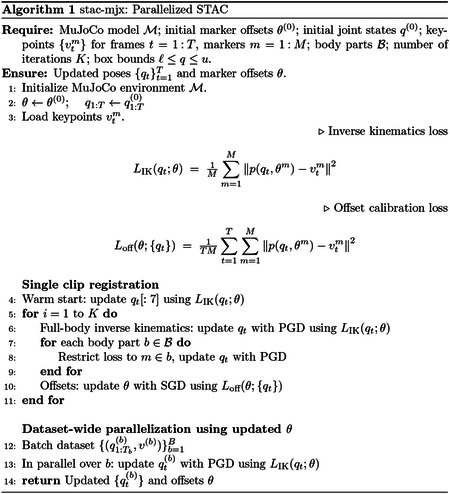


#### 4.3.1 Smoothing Edge Effects

We used **stac-mjx** on a 3 hour continuous session to evaluate our trained policy on long unseen clips. In this scenario, **stac-mjx** produced small but noticeable edge effects when stitching together segments that were independently processed. To account for this, we include a feature in **stac-mjx** to handle long continuous data by processing overlapping segments, then blending the overlapping region using a crossfade procedure:

Let $a,b\in R^{O\times D}$ be the two overlapping segments.

For j=0,…,O-1, define the normalized position:

$$x_{j}=\frac{j}{O-1}.$$

Using a fixed center c=0.5 and steepness s=10.0, the sigmoid mixing weight is:

$$m_{j}=\frac{1}{2}\left(1+\text{tanh}\left(\frac{10\left(x_{j}-0.5\right)}{2}\right)\right).$$

The crossfaded output CF(a,b) is defined elementwise by:

$$\text{CF}(a,b{)}_{j,d}=\left(1-m_{j}\right)a_{j,d}+m_{j}b_{j,d},\;j=0,\ldots ,O-1,\;d=1,\ldots ,D.$$

This procedure, in addition to tuning optimization parameters, results in negligible edge effects.

### 4.4 Deep Reinforcement Learning for motion imitation

#### 4.4.1 Parallelized training

On-policy reinforcement learning algorithms are generally divided into two phases: data collection and policy updates. Policy updates consist of performing backpropagation on the neural networks, which are housed on the GPU. Data collection from physics environments consists of policy inference, simulating steps, and reward/observation calculation. This phase is most commonly performed on the CPU [75–79] as popular physics engines such as MuJoCo are CPU-bounded. By leveraging MJX, we overcome the data transfer problem, and also substantially improve simulation speed through the parallel environments—thousands of environments can run on a single GPU workstation/cluster node. Parallelizing CPU-based simulation is also possible, but typically requires provisioning additional cluster nodes, high-bandwidth interconnects, and a nontrivial distributed-training setup (job schedulers, networking and firewall configuration, synchronization, and data movement). Achieving the necessary throughput with CPUs would ordinarily require multiple server-grade machines, whereas a single GPU can do the same job with far less operational overhead.

#### 4.4.2 Task Environment

##### Input Observations

The proprioceptive observations are sampled at each timestep from the physics environment and are provided both as feedback to the policy’s decoder and used to calculate the reference observations. They are defined as $s_{t}^{p}≜\left(\theta_{t},\omega_{t},\tau_{t},z_{t},\phi_{t},e_{t}\right)$ where $\theta_{t}$ are the joint angles, $\omega_{t}$ are the joint angular velocities, $\tau_{t}$ are the applied joint torques, $z_{t}$ is the root height above the ground, $\phi_{t}$ is the direction of the global z-axis relative to the agent (akin to a vestibular sense), and $e_{t}$ are the relative positions of the end effectors rotated to the egocentric frame.

The reference observations are kinematic features of the next t frames in the target trajectory, given in egocentric coordinates and supplied as input to the policy’s encoder. The reference observations are defined as $s_{t}^{g}≜\left(\Delta r_{t},\Delta \varphi_{t},\Delta \theta_{t},\Delta x_{t}\right)$ where $\Delta r_{t}$ is the difference between the agent and the target’s root position rotated to the agent’s egocentric frame, $\Delta \varphi_{t}$ is the difference between the agent and the target’s quaternions, $\Delta \theta_{t}$ is the difference between the agent and the target’s joint angles, and $\Delta x_{t}$ is the difference between the agent and the target’s local body positions in cartesian coordinates rotated to the agent’s egocentric frame. We compute the reference observations for 5 time steps into the future, allowing the agent’s policy to “look-ahead”. We find that the choice of this trajectory length affects the model’s ability to generalize to longer motion clips (Supplementary Fig. 6).

The observations are normalized to have μ≈0 and $\sigma^{2}\approx 1:{\overset{ˆ}{o}}_{t}=\left(o_{t}-\mu \right)/\sigma$. Welford’s online algorithm [80] is used to maintain these statistics efficiently. The running statistics are updated after every data collection round from the environment, and are maintained throughout training

##### Taking actions

Each environment step takes an action vector which maps to a control signal for each actuator in the animal model. The simulation is driven forward for a fixed timestep using this control signal to actuate the body. The duration of this timestep determines the policy’s action rate, which can be different for each animal model; this is defined in the environment configuration.

##### Reward

Similar to previous work [21, 25, 33], we decompose the objective of our rat and fly into four complementary reward terms to train the motion imitation policy. Together, they provide a dense reward signal representing the correct pose of the animal. At time t, the instantaneous reward is a weighted sum

$$r_{t}=\lambda_{\text{pos}}\;r_{t}^{\text{pos}}+\lambda_{\text{quat}}{\;r}_{t}^{\text{quat}}+\lambda_{\text{joint}\;}r_{t}^{\text{joint}}+\lambda_{\text{ee}}\;r_{t}^{\text{ee}},$$

where each $r_{t}^{\left(\cdot \right)}\in [0,1]$ increases as the learner better matches the registered reference trajectory. We define each of the reward terms as a gaussian kernel using L2 distance between the agent’s pose and the reference data:

$$r_{t}^{\left(.\right)}=\text{exp}\left(-\frac{1}{2\sigma_{\left(.\right)}^{2}}{\left\|q-q_{\text{ref}}\right\|}_{2}^{2}\right)$$

The σ term is configured for each reward term based on the distance scales.

1. **Position** ($r^{\text{pos}}$) penalizes the Cartesian displacement between the learner’s root/-global position and the reference, encoding *where* the body is in the arena.
2. **Quaternion / orientation** ($r^{\text{quat}}$) penalizes angular misalignment of headings, encoding *which way* the body is pointing.
3. **Joint** ($r^{\text{joint}}$) penalizes per-joint angle error, shaping local body configurations.
4. **End effector** ($r^{\text{ee}}$) penalizes distances between the learner’s distal effectors (e.g., hands, pretarsal claws) and the registered references, encouraging precise placement.

##### Termination criteria

Early termination criteria improve sample efficiency during training by avoiding training data in unrecoverable states. The environment terminates (leading to an automatic reset during training) if any of the following conditions are met:

- *Fall detection*. The agent’s root z-position in global cartesian coordinates falls outside a healthy range.
- *Maximum cartesian position error*. The agent’s root position in global cartesian coordinates sufficiently diverges from the reference.
- *Maximum orientation error*. The root quaternion of the agent sufficiently diverges from the reference.
- *Maximum joint pose error*. The joint angles of the agent in aggregate sufficiently diverges from the reference.

The environment is also reset once it reaches the end of the reference motion trajectory clip.

#### 4.4.3 Training with deep reinforcement learning

We employ Proximal Policy Optimization [39] as our reinforcement learning algorithm, with several modifications tailored for trajectory imitation learning. Our PPO implementation draws heavily from the Brax framework [81] while using our encoder-decoder policy architecture to compress motor intentions into a regularized stochastic latent space.

##### Problem Formalization

We can formalize the tracking objective as a Markov Decision Process (MDP) defined by the tuple ℳ=⟨𝒮,𝒜,𝒯,ℛ,γ⟩ containing states, actions, transition dynamics, reward function, and discount factor. The physics environment determines the state $s_{t}^{p}\in 𝒮$ and transition dynamics 𝒯. The policy $\pi_{\text{track}}$ computes the per-step action $a_{t}\in 𝒜$. The reward function computes the imitation reward, $r_{t}$, for the policy as a function of the simulation state, $s_{t}^{p}$ and the reference motion, $s_{t}^{\text{mocap}}:r_{t}=ℛ\left(s_{t}^{p},s_{t}^{\text{mocap}}\right)$. The objective of the policy is to maximum the discounted return $E\left[\sum_{t=1}^{T}\gamma^{t-1}r_{t}\right]$.

##### Policy network

Similar to prior work [21], our policy has an encoder-decoder architecture with a variational bottleneck in between, akin to a Variational Autoencoder [82]. Both the encoder and decoder are MLPs. The encoder, $ℰ\left(z_{t}|s_{t:t^{\prime }}^{g}\right)=𝒩\left(z_{t}|\mu_{t}^{e},\sigma_{t}^{e}\right)$, receives a context window of reference observations as input and outputs the mean and logvariance of a diagonal Gaussian distribution. The reparameterization trick [82] is used to sample this distribution. The sampled latent vector, $z_{t}$, is passed as input to the decoder along with the proprioceptive observation from the environment, $𝒟\left(a_{t}|s_{t}^{p},z_{t}\right)=\text{tanh}\left[𝒩\left(a_{t}|\mu_{t}^{a},\sigma_{t}^{a}\right)\right]$, and outputs a parametric action distribution defined as a normal distribution followed by a hyperbolic tangent to bound the actions within [−1, 1]. At inference, the mean of both the encoder and decoder is used to generate actions. The weights of the networks are initialized using LeCun Uniform [83]. Both the encoder and decoder use SiLU activations [84] followed by layer normalization [85]. Network layer configurations are detailed in the supplement.

##### Latent space regularization

In order to regularize the intention space of the variational bottleneck, ℰ(z), we enforce an Order 1 Autoregressive (AR(1)) prior [34, 86] through a Kullback-Leibler divergence (KL) loss. Compared to the standard isotropic Gaussian prior in VAEs, this prior encourages temporally correlated latents over action sequences, with each latent being conditioned on the previous one. This form of regularization has been shown to be important when applying the decoder to downstream tasks by making it amenable to RL exploration through stochastic actions [87]. With no previous latent, the first intention is regularized to be close to a standard Gaussian distribution, while subsequent intentions are maintained close to the previous intentions:

$$\text{First}\;\text{latent}:\;AR(1)\left[ℰ\left(z_{0}\right)\right]=KL\left(ℰ\left(z_{0}\right)\|𝒩(0,I)\right)$$

$$\text{Subsequent}\;\text{latents}:\;AR(1)\left[ℰ\left(z_{t}\right)\right]=KL\left(ℰ\left(z_{t}\right)\|𝒩\left(\alpha \cdot z_{t-1},\sigma^{2}I\right)\right)$$

where α=0.95 and $\sigma^{2}=1-\alpha^{2}=0.0975$. During training, we employ a linear ramp-up scheduler for the AR(1) loss.

##### Value network

We employ a standard feed-forward MLP network as our critic. The network receives the complete observation (proprioceptive and reference observations) as input. The output is a single scalar value representing the estimated value of the current state. This network uses the LeCun Uniform initialization and employs ReLU activations.

##### Environment initialization and data collection

We use reference motion clips with lengths of $L_{\text{clip}}$ frames sampled at a rate of $f_{\text{mocap}}$. We initialize $N_{\text{envs}}$ environments in parallel using randomly chosen clips. Each environment begins at a random frame chosen from the first $n_{\text{init}}$ frames in each clip. A small amount of noise $\sigma_{\text{noise}}$ is added to both the pose and the velocity of the first frame to prevent overfitting to exact initial conditions. We define the length of a full episode to be $T_{\text{episode}}$.

After initialization, we define one iteration of the data collection phase as the collection of $N_{\text{minibatch}}$ number of mini-batches, each containing $B_{\text{batch}}$ trajectories spanning $L_{\text{unroll}}$ frames. We define one round of data collection as collecting $L_{\text{unroll}}$ frames of data from each of the $N_{\text{envs}}$ environments. $L_{\text{unroll}}$ must be large enough to capture coherent temporal dynamics, but short enough to ensure training stability. If $L_{\text{unroll}}$ is too small, Generalized Advantage Estimation (GAE) cannot leverage rewards from enough timesteps to be effective. If $L_{\text{unroll}}$ is too large, we obtain less stable gradient estimates, risking training destabilization. As such, multiple rounds of data collection may be needed to fill the mini-batch quota before performing a gradient update. The data collection process is outlined in algorithm 2.


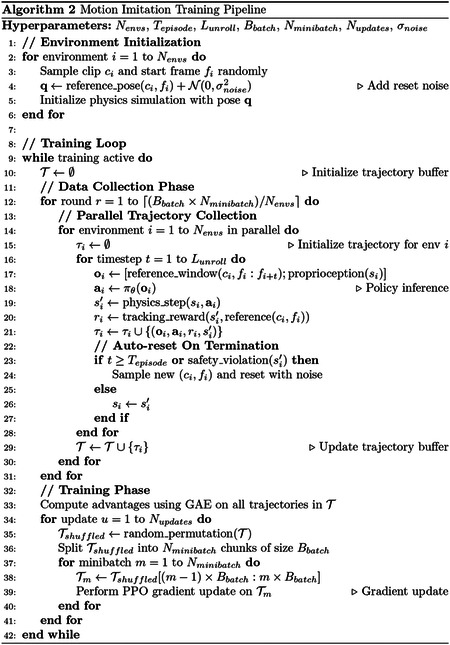


#### 4.4.4 Transfer learning via a frozen MIMIC-MJX decoder

##### Pretraining

First, we training an encoder–decoder policy on motion imitation (Fig. 5a, top). An encoder $q_{\phi }\left(z_{t}|s_{t}^{g}\right)$ maps the guide/target state sequence $s_{t}^{g}$ (from demonstrations) to a Gaussian over latent commands with parameters ($\mu_{t},\sigma_{t}$). An AR(1) prior imposes temporal smoothness on the latents, and a decoder, $p_{\theta }\left(a_{t}|z_{t},s_{t}^{p}\right)$, maps the latent command $z_{t}$ together with the current proprioceptive state $s_{t}^{p}$ to an action distribution. Actions are executed in physics to produce $s_{t+1}^{p}$. After this stage, θ defines a reusable *skill decoder* that interprets latent commands into body-specific motor outputs.

##### Policy transfer instantiation

To reuse the pre-trained controller on new tasks, we *freeze* the decoder parameters θ (denoted by the snowflake in Fig. 5a, bottom) and replace the encoder with a task policy $\pi_{\psi }$:

$$z_{t}=\pi_{\psi }\left(o_{t}\right),\;a_{t}\sim p_{\theta }\left(a_{t}|z_{t},s_{t}^{p}\right),$$

where $o_{t}$ is any task-specific observation (e.g., vision features, goals, rewards). Only ψ is updated during transfer; θ remains fixed.

In practice, we first load the pre-trained decoder $p_{\theta }\left(a_{t}|z_{t},s_{t}^{p}\right)$ and keep its parameters fixed. We then construct a task policy $\pi_{\psi }$ that maps task observations $o_{t}$ to latent commands $z_{t}$ with the same dimensionality used during pretraining. The decoder continues to receive the same proprioceptive input $s_{t}^{p}$ as in pretraining (for the same body model). Training proceeds by rolling out in simulation: the task policy produces latents, the frozen decoder converts them to actions, rewards are computed for the task, and only ψ is updated (e.g., via reinforcement learning) while θ remains fixed.

This modular interface makes the latent space $z_{t}$ effectively task-agnostic—structured during imitation pretraining by the decoder and then driven by a lightweight task policy for rapid transfer.

### 4.5 Bowl Escape Environment

The overall objective of the bowl escape environment is that the agent needs to learn a way to escape from the center of the bowl as far as possible. Notably, the reward design itself is not concerned about a specific behavior that you use to escape the bowl, since the reward function only cares about the location of the agents.

#### 4.5.1 State Space and Task Overview

The agent (a torque-controlled body with a floating base) starts near the center of a concave heightfield (the “bowl”) and is rewarded for reaching the rim while staying upright and moving near a target speed. Because MJX does not currently support collisions between height fields and ellipsoid geometries, we replaced the rat’s ellipsoid foot geoms with boxes for this transfer task. The ground is a heightfield $H\in [0,1{]}^{N\times N}$ scaled in world coordinates by horizontal scale h>0 (half–width) and vertical scale v>0.

##### World/grid mapping.

Let world planar coordinates be $(x,y)\in [-h,h{]}^{2}$. Define normalized texture coordinates

$$u=\frac{x+h}{2h},\;v=\frac{y+h}{2h}\;(u,v\in [0,1]),$$

and corresponding grid indices

$$j=\left\lfloor u\left(N-1\right)\right\rfloor ,\;i=\left\lfloor v\left(N-1\right)\right\rfloor ,\;(i,j)\in \{0,\ldots ,N-1{\}}^{2}.$$

The world’s ground height is

(1) $$z_{\text{ground}}\left(x,y\right)=v\;H[i,j].$$

The grid spacing in world units is $\Delta =\frac{2h}{N-1}$.

#### 4.5.2 Bowl Geometry

We construct a smooth concave “Gaussian bowl” and add controllable roughness via periodic 2D Perlin noise. A radial gate blends out the noise near the origin to ensure a clean launch zone.

##### Base Gaussian Depression

On a normalized square $(\xi ,\eta )\in [-1,1{]}^{2}$ (linearly mapped from grid indices), define the radially symmetric depression

(2) $$B\left(\xi ,\eta ;\sigma ,A\right)=A\;\text{exp}\left(-\frac{\xi^{2}+\eta^{2}}{2\sigma^{2}}\right),\;\sigma >0,\;A<0,$$

sampled at N×N points to obtain $B\in R^{N\times N}$. Larger |A| deepens the bowl; smaller σ sharpens it.

##### Tileable 2D Perlin Noise

Let $R_{x},R_{y}\in N$ be the desired number of periods across the domain. Construct a rectified integer lattice

$$ℒ=\left\{\left(p,q\right)|p\in \left\{0,\ldots ,R_{x}\right\},\;q\in \left\{0,\ldots ,R_{y}\right\}\right\},$$

with periodic boundary conditions so that gradients at p=0 and $p=R_{x}$ (and analogously in q) match. Assign each node (p,q)∈ℒ a unit gradient $g_{pq}\in R^{2}$.

For a continuous coordinate $(\tilde{u},\tilde{v})\in [0,1{]}^{2}$, scale by periods,

$$\overset{‾}{u}=R_{x}\;\tilde{u},\;\overset{‾}{v}=R_{y}\tilde{v},$$

and let $p=\lfloor \overset{‾}{u}\rfloor ,q=\lfloor \overset{‾}{v}\rfloor$, with local cell coordinates

$$t_{x}=\overset{‾}{u}-p,\;t_{y}=\overset{‾}{v}-q\;\left(t_{x},t_{y}\in [0,1]\right).$$

Define corner dot products

(3) $$n_{00}=\left\langle g_{p,q},\left(t_{x},t_{y}\right)\right\rangle ,\;n_{10}=\left\langle g_{p+1,q},\left(t_{x}-1,t_{y}\right)\right\rangle ,$$

(4) $$n_{01}=\left\langle g_{p,q+1},\left(t_{x},t_{y}-1\right)\right\rangle ,\;n_{11}=\left\langle g_{p+1,q+1},\left(t_{x}-1,t_{y}-1\right)\right\rangle ,$$

with indices taken modulo ($R_{x},R_{y}$) for tileability. Using the quintic fade $f\left(s\right)=6s^{5}-15s^{4}+10s^{3}$, interpolate

(5) $${\tilde{n}}_{0}=\left(1-f\left(t_{x}\right)\right)n_{00}+f\left(t_{x}\right)n_{10},$$

(6) $${\tilde{n}}_{1}=\left(1-f\left(t_{x}\right)\right)n_{01}+f\left(t_{x}\right)n_{11},$$

(7) $$P(\tilde{u},\tilde{v})=\left(1-f\left(t_{y}\right)\right){\tilde{n}}_{0}+f\left(t_{y}\right){\tilde{n}}_{1}.$$

The raw Perlin field P has zero mean and bounded variance; we optionally scale and shift to [0, 1] by

(8) $$\tilde{P}=\frac{P-\text{min}(P)}{\text{max}(P)-\text{min}(P)}.$$

##### Noise Gating and Normalization

Let $c=\frac{N-1}{2}$ be the grid center and $r_{ij}=\sqrt{(i-c{)}^{2}+(j-c{)}^{2}}$ a discrete radius. Choose inner/outer gating radii $0\leq r_{\text{in}}<r_{\text{out}}\leq \sqrt{2}\frac{N-1}{2}$. Define a smooth radial weight

(9) $$w_{ij}=\text{smoothstep}\left(\frac{r_{ij}-r_{\text{in}}}{r_{\text{out}}-r_{\text{in}}}\right),\;\text{smoothstep}\left(s\right)=\left\{\begin{array}{ll} 0, & s\leq 0,\\ 3s^{2}-2s^{3}, & 0<s<1\\ 1, & s\geq 1. \end{array}\right.,$$

Given a noise amplitude λ≥0, the raw height is

(10) $$H_{0}=\left(1-w\right)⊙B+w⊙\left(B+\lambda \tilde{P}\right),$$

where ⊙ denotes element-wise multiplication and w the matrix of $w_{ij}$. Finally, normalize to [0,1]:

(11) $$H=\frac{H_{0}-\text{min}\left(H_{0}\right)}{\text{max}\left(H_{0}\right)-\text{min}\left(H_{0}\right)}\in [0,1{]}^{N\times N}.$$

##### Surface Tangents and Normal

Finite differences on the grid give

(12) $${\left.\partial_{x}H\right|}_{ij}\approx \frac{H[i,j+1]-H[i,j-1]}{2},{\left.{\;\partial }_{y}H\right|}_{ij}\approx \frac{H[i+1,j]-H[i-1,j]}{2}.$$

In world units (with vertical scale v and horizontal step Δ), the ground surface at (i,j) has tangents

(13) $$t_{x}=\left(\Delta ,0,v\;\partial_{x}H\right),\;t_{y}=\left(0,\Delta ,v\;\partial_{y}H\right),$$

and the unit normal

(14) $$\overset{ˆ}{n}=\frac{t_{x}\times t_{y}}{\left\|t_{x}\times t_{y}\right\|}.$$

#### 4.5.3 Observations

At time t, the policy input concatenates a task–specific vector $o_{t}^{\text{task}}$ and a proprioceptive vector $o_{t}^{\text{prop}}$:

$$o_{t}=\left[o_{t}^{\text{task}}\|o_{t}^{\text{prop}}\right].$$

##### Task–specific observations.

(15) $$o_{t}^{\text{task}}=\left[a_{t-1},o^{\text{base}},o^{\text{kin}},o^{\text{touch}},o^{\text{orig}}\right],$$

where $a_{t-1}$ is the previous action; $o^{\text{base}}$ contains body–frame base orientation and linear/angular velocities; $o^{\text{kin}}$ collects joint angles/velocities; $o^{\text{touch}}$ encodes contact/tactile readings; and $o^{\text{orig}}$ provides the agent pose relative to the bowl origin (e.g., planar displacement and heading in the torso frame).

##### Proprioceptive observations.

Let q and $\dot{q}$ be generalized coordinates and velocities, excluding the floating base entries. With actuator signals $\tau_{\text{act}}$ and selected end–effector positions expressed in the torso frame,

(16) $$o_{t}^{\text{prop}}=\left[q_{7:},{\dot{q}}_{6:},\tau_{\text{act}},p_{\text{EE}},o^{\text{kin}}\right].$$

#### 4.5.4 Reward and Termination

Let the torso planar position be $\left(x_{t},y_{t}\right)$ and $\rho_{t}=\sqrt{x_{t}^{2}+y_{t}^{2}}$. The bowl half–width is h.

##### Reward Scaling

For a scalar s and target interval [a,b] (with a≤b), define a piecewise–linear tolerance:

(17a) $$\begin{array}{rr} {\text{tol}}_{\text{lin}}(s;[a,b],m) & =\left\{\begin{array}{ll} 0, & s\leq 0\\ \text{min}\left(1,\frac{s}{m}\right), & a=b=m,\\ \text{clip}\left(\frac{s-a}{b-a},0,1\right), & a<b, \end{array}\right. \end{array}$$

(17b) clip(x,0,1)=min(1,max(0,x)).

##### Spawn Launchpad Smoothing

A radius–to–rim shaping that ramps from 0 at the center to 1 by the rim:

(18) $$r_{\text{esc}}(t)=\text{min}\left(1,\frac{\rho_{t}}{h}\right).$$

##### Uprightness

Let $\overset{ˆ}{z}$ be the world up-axis, and let $z_{\text{torso}},z_{\text{head}}$ be the local z-axes (third rotation columns) of torso and head. With allowable tilt θ (typically $\theta =0^{\circ }$),

(19) $$r_{\text{up}}\left(t\right)=\frac{1}{2}{\text{tol}}_{\text{lin}}\left(\left\langle z_{\text{torso}},\overset{ˆ}{z}\right\rangle ;\left[\text{cos}\;\theta ,\infty \right),1+\text{cos}\;\theta \right)+\frac{1}{2}{\text{tol}}_{\text{lin}}\left(\left\langle z_{\text{head}},\overset{ˆ}{z}\right\rangle ;\left[\text{cos}\;\theta ,\infty \right),1+\text{cos}\;\theta \right).$$

##### Speed Setpoint

Let $v_{t}=\left\|{\vec{v}}_{\text{torso}}(t)\right\|$ be the torso linear speed and $v^{⋆}>0$ a target speed. A triangular peak at $v^{⋆}$ is

(20) $$r_{\text{spd}}\left(t\right)={\text{tol}}_{\text{lin}}\left(v_{t};\left[v^{⋆},v^{⋆}\right],v^{⋆}\right),$$

which equals 1 at $v_{t}=v^{⋆}$ and decreases linearly to 0 at $v_{t}\in \left\{0,2v^{⋆}\right\}$.

##### Total Reward


(21) $$r_{t}=\underset{\text{reach}\;\text{rim}\;\text{while}\;\text{upright}}{\underbrace{r_{\text{esc}}(t)\cdot r_{\text{up}}(t)}}+\underset{\text{move}\;\text{near}\;\text{target}\;\text{speed}}{\underbrace{r_{\text{spd}}(t)}}$$

##### Termination

Let $z_{t}$ be the torso height and $z_{\text{ground}}\left(x_{t},y_{t}\right)$ the terrain height below the torso. The episode fails if

(22) $$z_{t}\leq z_{\text{ground}}\left(x_{t},y_{t}\right)+\varepsilon ,\;\varepsilon \approx 0.03\;\text{m}.$$

##### Environment Parameters


N=128,h=2.0,v=0.2,

σ=1.25,A=-10,λ∈[0,1],

$$R_{x}=R_{y}=8,\;r_{\text{in}}=0.05N,{\;r}_{\text{out}}=0.25N,$$

$$v^{⋆}=0.75\;\text{m}/\text{s},\;\text{horizon}\approx 1500\;\text{control}\;\text{steps}.$$

### 4.6 Gait Analysis

We segment gait cycles by employing swing-stance transitions, detected as collisions between the rat’s hands and the environment’s ground. A gait cycle is defined from the beginning of a stance until the beginning of the next stance. The left forepaw was chosen to determine the boundaries of the gait cycles. We achieve phase normalization of joint angles by resampling all phase-locked gait cycles to 100 time points to represent the percentage of the gait cycle progression using cubic interpolation.

### 4.7 Latent Space Analysis

#### 4.7.1 Mouse arm reaching

To analyze the representational geometry of the network, we applied principal component analysis (PCA) to the intention bottleneck and to each of the three hidden layers of the encoder and decoder. For each layer, activations with dimensionality (clips, timesteps, 512) were reshaped into a two-dimensional matrix of shape (clips × timesteps, 512) and PCA was performed. The first three principal components were retained, and the transformed data were reshaped back into (clips, timesteps, 3) to preserve temporal and clip structure. The same procedure was applied to the intention representations, resulting in the top three principal components. For each analysis, the proportion of variance explained by the first three components was recorded to quantify the representational compression achieved by PCA. To enable joint visualization of neural and behavioral dynamics, the three components were concatenated with a behavioral covariate (the joint angle $q_{\text{pose}}$), producing arrays of shape (clips, timesteps, 4). The 3D visualization of the top 3 PCs was visualized for the latent bottleneck and each layer of the decoder (Fig. 5c).

#### 4.7.2 Fly walking

Using a trained policy on a dataset of fly walking, with a latent dimensionality of 60, we recorded network activations during each clip of walking at various speeds. We represented each trajectory frame by sampling the latent space activity with a time-delay embedding with a half-window of 30ms, resulting in a 31 frame window of latent space activations with a total of 1860 dimensions (60 * 31). To calculate the speed of each clip in Fig. 5d, we calculated the instantaneous speed between each frame, averaging over the clip to get the final value.

## Supplementary Material

1

## Data Availability

We have open-sourced all the data used in this work. This includes data that is already publicly available, as well as data collected by our team. Data that was already publicly available has been cited in this work. Model weights are available on Hugging Face at https://huggingface.co/talmolab/MIMIC-MJX and sample configuration files and datasets are available at https://huggingface.co/datasets/talmolab/MIMIC-MJX.

## Code Availability.

Our code is open-sourced and available at https://github.com/talmolab/stac-mjx and https://github.com/talmolab/track-mjx. Documentation, tutorials, and usage guides are available on the documentation page at https://mimic-mjx.talmolab.org.
