# Supplementary material for: MIMIC-MJX: Neuromechanical Emulation of Animal Behavior
Source: ArXiv. 2025 Dec 2:arXiv:2511.20532v2. Preprint. [Version 2] (PMC12676414)
Supplement: 1 [file NIHPP2511.20532V2-supplement-1.pdf]

5 Supplementary Information

The online version of the article contains supplementary material added below.

Supplementary Figures.

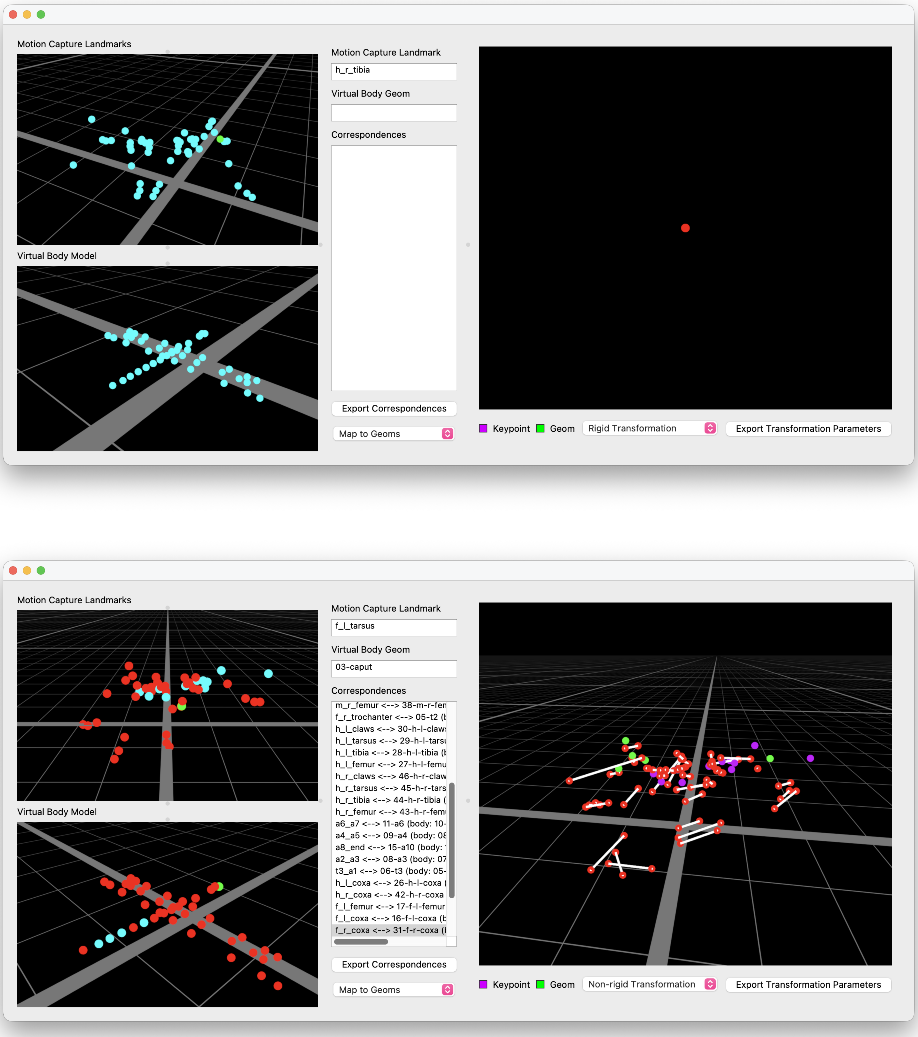

Supplementary Fig. 1: Screenshots of the STAC Keypoint Correspondence user interface. Top: Initial interface displaying pose tracking landmarks (blue) and the virtual body model. Bottom: Interface after establishing correspondences. Red dots indicate established correspondences; blue dots indicate unmatched points.

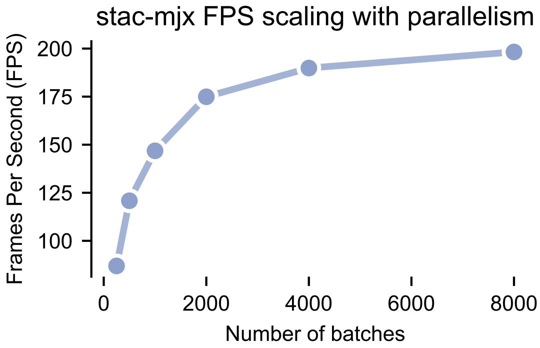

Supplementary Fig. 2: **stac-mjx** frame processing speed as a function of the number of parallel batches. The plot shows frames per second (FPS) when processing 360,000 total frames across different batch configurations.

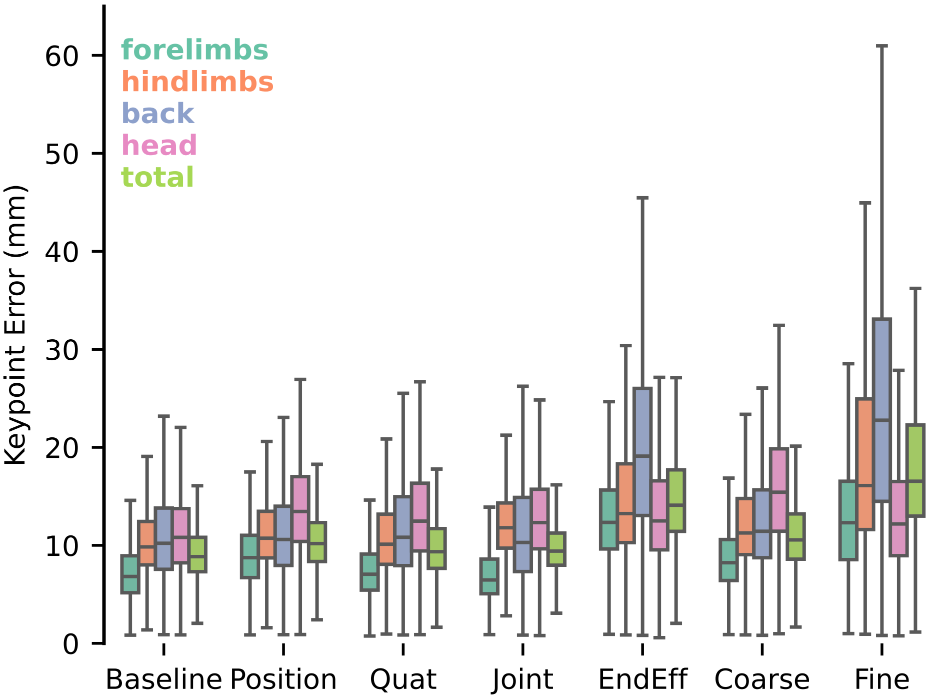

Supplementary Fig. 3: Ablation studies on various reward terms of the rat. Keypoint error distributions are shown as between **track-mjx**-reproduced trajectories and **stac-mjx**-registered reference.

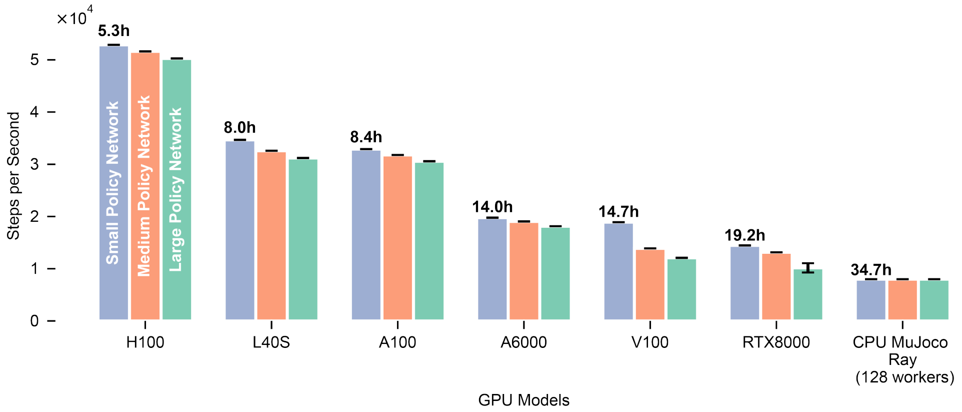

Supplementary Fig. 4: SPS for the rat environment on different hardware: H100, L40S, A100, A6000, V100, RTX8000, and a baseline CPU MuJoCo implementation.

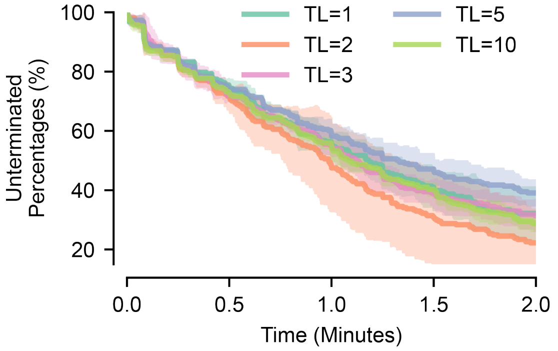

Supplementary Fig. 5: Evaluation of continuous tracking performance for variable trajectory length (TL) on a held-out dataset.

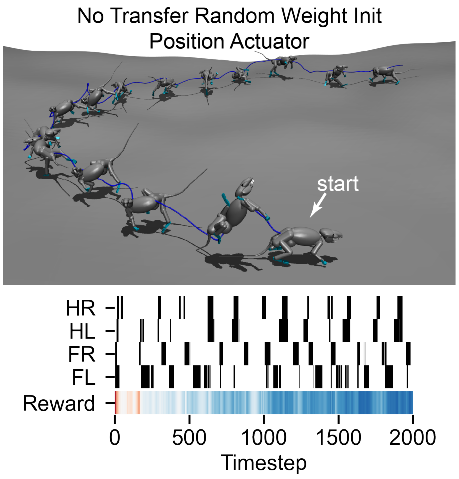

Supplementary Fig. 6: Bowl escape task solved by a randomly initialized policy using position actuators. Top: rendered trajectory. Bottom: end-effector contact timing and cumulative reward. The policy achieves task success but with unnatural gait. Paw abbreviations HR: hind-right, HL: hind-left, FR: fore-right, FL: fore-left.

Supplementary Tables.

Table 1: Imitation training config parameters for each animal body model.

**PPO Params**	**Rat**	**Fly**	**Worm**	**Mouse Arm**	**Stick Insect**
num envs	4096	4096	4096	4096	4096
batch size	1024	1024	1024	1024	1024
num minibatches	16	16	16	8	16
learning rate	0.0001	0.0001	0.0001	0.0001	0.0001
clipping epsilon	0.2	0.2	0.2	0.2	0.2
discounting	0.95	0.95	0.95	0.95	0.97
entropy cost	0.01	0.01	0.002	0.001	0.0001
unroll length	20	20	20	20	20
kl weight	0.1	0.1	0.0001	0.00001	0.1
**Network Params**					
encoder layer sizes	[512,256,256]	[256,256]	[512,512,512]	[512,512,512]	[512,512,512]
decoder layer sizes	[512,512,256,256]	[256,256]	[512,512,512]	[512,512,512]	[512,512,512,256]
critic layer sizes	[512,512,256,256]	[256,256]	[512,512,512]	[512,512,512]	[512,512,256]
intention size	60	60	8	4	60
**Sim Params**					
sim dt	0.002	0.0002	0.005	0.00125	0.002
ctrl dt (sim_dt * steps...)	0.01	0.002	0.1	0.0025	0.01
solver	CG	CG	Newton	CG	CG
iterations	5	4	4	6	6
ls iterations	5	4	4	6	6
**Reward Params**					
pos exp scale	400	400	0.1	0.0	40000
quat exp scale	4	4	30	0.0	4
joint exp scale	0.25	0.25	1	0.2	0.25
end eff exp scale	500	100	0.3	0.0	50000
body pos exp scale	0.0	8	0.5	0.0	8
joint vel exp scale	0.0	0.5	1	0.0	0.5
pos weight	1	1	1	0.0	1
quat weight	1	1	1	0.0	1
joint weight	1	1	2	5	1
end eff weight	1	1	1	0.0	1
body pos weight	0.0	0.0	1	0.0	0.0
joint vel weight	0.0	0.0	3	0.0	0.0
control cost	0.02	0.02	0.0	0.15	0.02
control difference cost	0.02	0.1	0.0	0.0	0.02
energy cost	0.01	0.001	0.0	0.01	0.001
variance cost	0.01	0.0	0.0	0.0	0.0
variance window	50	0.0	0.0	0.0	0.0

Supplementary Movies. Available online at: https://mimic-mjx.talmolab.org

1. **Supp. Movie 1**: The video shows MIMIC-MJX motion capture registration and replay demonstrated across diverse morphologies: rat, fly, mouse arm, worm, and stick insect.
2. **Supp. Movie 2**: The video shows transfer learning performance in the rat bowl-escape task, comparing agents with and without pretrained decoder initialization.
